# Methyl 6′-amino-5′-cyano-2′-methyl-2-oxospiro­[indoline-3,4′-pyran]-3′-carboxyl­ate

**DOI:** 10.1107/S1600536810053274

**Published:** 2010-12-24

**Authors:** Song-Lei Zhu, Ting Liu

**Affiliations:** a Department of Chemistry, Xuzhou Medical College, Xuzhou 221004, People’s Republic of China

## Abstract

In the mol­ecule of the title compound, C_16_H_13_N_3_O_4_, the atoms of the spiro pyran ring are nearly planar with a maximum deviation of 0.095 (2) Å. The indole and pyran rings are oriented at a dihedral angle of 87.3 (9)°. In the crystal, mol­ecules are linked by inter­molecular N—H⋯N and N—H⋯O hydrogen bonds.

## Related literature

For the indole nucleus, see: Da-Silva *et al.* (2001 ▶). Compounds carrying the indole moiety exhibit anti­bacterial and fungicidal activity, see: Joshi & Chand (1982 ▶). Spiro­oxindole ring systems are found in a number of alkaloids like horsifiline, spiro­tryprostatin and elacomine, see: Abdel-Rahman *et al.* (2004 ▶). For our work on the preparation of heterocyclic compounds involving indole derivatives, see: Zhu *et al.* (2007 ▶).
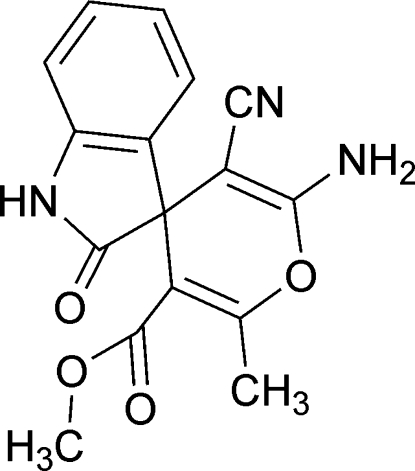


## Experimental

### 

#### Crystal data

C_16_H_13_N_3_O_4_
*M* *_r_* = 311.29
Monoclinic, 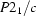

*a* = 15.0260 (15) Å
*b* = 10.0614 (11) Å
*c* = 10.4862 (12) Å
β = 105.956 (1)°
*V* = 1524.3 (3) Å^3^
*Z* = 4
Mo *K*α radiation
μ = 0.10 mm^−1^
*T* = 298 K
0.48 × 0.46 × 0.30 mm

#### Data collection

Bruker SMART CCD area-detector diffractometer
Absorption correction: multi-scan (*SADABS*; Sheldrick, 1996 ▶) *T*_min_ = 0.954, *T*_max_ = 0.971
7311 measured reflections
2686 independent reflections
1868 reflections with *I* > 2σ(*I*)
*R*_int_ = 0.032

#### Refinement

*R*[*F* ^2^ > 2σ(*F* ^2^)] = 0.045
*wR*(*F* ^2^) = 0.127
*S* = 1.04
2686 reflections
210 parameters
H-atom parameters constrained
Δρ_max_ = 0.21 e Å^−3^
Δρ_min_ = −0.26 e Å^−3^

### 

Data collection: *SMART* (Bruker, 2004 ▶); cell refinement: *SAINT* (Bruker, 2004 ▶); data reduction: *SAINT*; program(s) used to solve structure: *SHELXS97* (Sheldrick, 2008 ▶); program(s) used to refine structure: *SHELXL97* (Sheldrick, 2008 ▶); molecular graphics: *SHELXTL* (Sheldrick, 2008 ▶); software used to prepare material for publication: *SHELXTL*.

## Supplementary Material

Crystal structure: contains datablocks global, I. DOI: 10.1107/S1600536810053274/bx2334sup1.cif

Structure factors: contains datablocks I. DOI: 10.1107/S1600536810053274/bx2334Isup2.hkl

Additional supplementary materials:  crystallographic information; 3D view; checkCIF report

## Figures and Tables

**Table 1:** Hydrogen-bond geometry (Å, °)

*D*—H⋯*A*	*D*—H	H⋯*A*	*D*⋯*A*	*D*—H⋯*A*
N1—H1⋯N3^i^	0.86	2.09	2.928 (3)	165
N2—H2*A*⋯O1^ii^	0.86	2.17	2.925 (2)	147
N2—H2*B*⋯O1^iii^	0.86	2.34	3.022 (2)	136

Symmetry codes: (i) 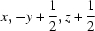; (ii) 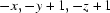; (iii) 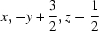.

**Fig. 1.**
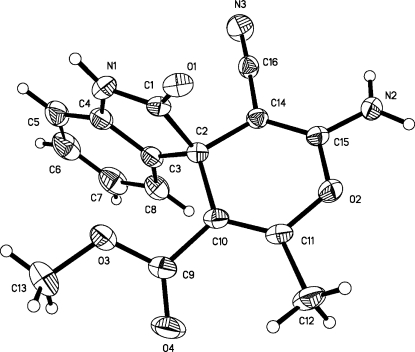
The molecular structure of the title molecule, with the atom-numbering scheme. Displacement ellipsoids are drawn at the 45% probability level.

**Fig. 2.**
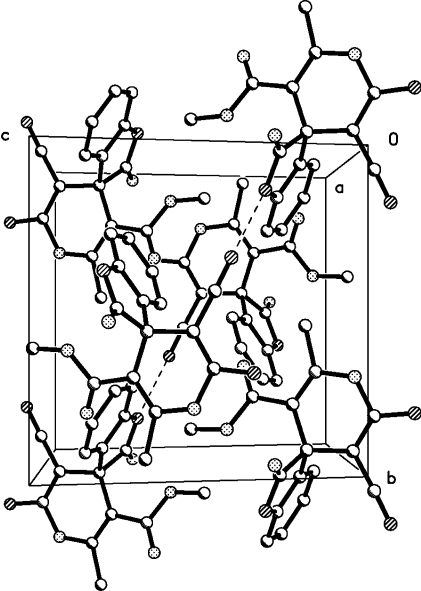
A packing diagram of (I). Hydrogen bonds are shown as dashed lines.

## supplementary crystallographic information

### Comment

The indole nucleus is the well known heterocycle (Da-Silva *et al.*, 2001). Compounds carrying the indole moiety exhibit antibacterial and fungicidal activities (Joshi & Chand, 1982). Spirooxindole ring systems are found in a number of alkaloids like horsifiline, spirotryprostatin and elacomine (Abdel-Rahman *et al.*, 2004). As a part of our programme devoted to the preparation of heterocyclic compounds involving indole derivatives (Zhu *et al.*, 2007), we have synthesized a series of spirooxindoles *via* reactions of isatins together with malononitrile and methyl 3-oxobutanoate in water. We report herein the crystal structure of the title compound, (I), (Fig. 1). The new formed spiro pyran ring A (O2/C2/C10/C11/C14/C15) adopts nearly planar conformation. The indole system and pyran ring are oriented at a dihedral angle of 87.3 (9)°. In the crystal structure, the molecules are linked by intermolecular N—H···N and N—H···O hydrogen bonds, Table 1, (Fig. 2).

### Experimental

Compound (I) was prepared by the reaction of isatin (1 mmol), malononitrile (1 mmol) and methyl 3-oxobutanoate (1 mmol) in water (5 ml). The reaction was catalyzed by TEBAC (triethylbenzylammonium chloride, 1 mmol). After stirring at 333 K for 5 h, the reaction mixture was cooled and washed with small amount of ethanol. The crude product was filtered and single crystals of the title compound were obtained from ethanol solution by slow evaporation at room temperature (yield; 82%, m.p. 535-536 K). Spectroscopic analysis: IR (KBr, ν , cm^-1^): 3458, 3362, 3217, 2212, 1729, 1628, 1464, 1376, 1284, 1210, 1057, 751, 676, 622. ^1^H NMR (400 MHz, DMSO-d_6_): 10.32 (s, 1H, NH), 7.21-7.25 (m, 3H, NH_2_ + ArH), 6.97-7.03 (m, 2H, ArH), 6.78 (d, J = 10.4 Hz, 1H, ArH), 3.76 (s, 3H, CH_3_), 2.25 (s, 3H, CH_3_).

### Refinement

H atoms were positioned geometrically, with N—H = 0.86 Å (for NH) and C—H = 0.93 and 0.96 Å for aromatic and methyl H, respectivly and constrained to ride on their parent atoms with U_iso_(H) = xU_eq_(C,N), where x = 1.5 for methyl H and x = 1.2 for all other H atoms.

### Crystal data

**Table:**

C_16_H_13_N_3_O_4_	*F*(000) = 648
*M**_r_* = 311.29	*D*_x_ = 1.357 Mg m^−^^3^
Monoclinic, *P*2_1_/*c*	Melting point = 535–536 K
Hall symbol: -P 2ybc	Mo *K*α radiation, λ = 0.71073 Å
*a* = 15.0260 (15) Å	Cell parameters from 2324 reflections
*b* = 10.0614 (11) Å	θ = 2.5–25.2°
*c* = 10.4862 (12) Å	µ = 0.10 mm^−^^1^
β = 105.956 (1)°	*T* = 298 K
*V* = 1524.3 (3) Å^3^	Block, colorless
*Z* = 4	0.48 × 0.46 × 0.30 mm

### Data collection

**Table:**

Bruker SMART CCD area-detector diffractometer	2686 independent reflections
Radiation source: fine-focus sealed tube	1868 reflections with *I* > 2σ(*I*)
graphite	*R*_int_ = 0.032
phi and ω scans	θ_max_ = 25.0°, θ_min_ = 1.4°
Absorption correction: multi-scan (*SADABS*; Sheldrick, 1996)	*h* = −17→17
*T*_min_ = 0.954, *T*_max_ = 0.971	*k* = −11→11
7311 measured reflections	*l* = −10→12

### Refinement

**Table:**

Refinement on *F*^2^	Primary atom site location: structure-invariant direct methods
Least-squares matrix: full	Secondary atom site location: difference Fourier map
*R*[*F*^2^ > 2σ(*F*^2^)] = 0.045	Hydrogen site location: inferred from neighbouring sites
*wR*(*F*^2^) = 0.127	H-atom parameters constrained
*S* = 1.04	*w* = 1/[σ^2^(*F*_o_^2^) + (0.0591*P*)^2^ + 0.418*P*] where *P* = (*F*_o_^2^ + 2*F*_c_^2^)/3
2686 reflections	(Δ/σ)_max_ < 0.001
210 parameters	Δρ_max_ = 0.21 e Å^−^^3^
0 restraints	Δρ_min_ = −0.26 e Å^−^^3^

### Special details

**Table:**

Geometry. All esds (except the esd in the dihedral angle between two l.s. planes) are estimated using the full covariance matrix. The cell esds are taken into account individually in the estimation of esds in distances, angles and torsion angles; correlations between esds in cell parameters are only used when they are defined by crystal symmetry. An approximate (isotropic) treatment of cell esds is used for estimating esds involving l.s. planes.
Refinement. Refinement of F^2^ against ALL reflections. The weighted R-factor wR and goodness of fit S are based on F^2^, conventional R-factors R are based on F, with F set to zero for negative F^2^. The threshold expression of F^2^ > 2sigma(F^2^) is used only for calculating R-factors(gt) etc. and is not relevant to the choice of reflections for refinement. R-factors based on F^2^ are statistically about twice as large as those based on F, and R- factors based on ALL data will be even larger.

### Fractional atomic coordinates and isotropic or equivalent isotropic displacement parameters (Å^2^)

**Table:**

	*x*	*y*	*z*	*U*_iso_*/*U*_eq_	
N1	0.19598 (12)	0.37685 (18)	0.79711 (19)	0.0494 (5)	
H1	0.1769	0.3191	0.8441	0.059*	
N2	0.04118 (12)	0.68602 (18)	0.34455 (18)	0.0465 (5)	
H2A	0.0244	0.6167	0.2958	0.056*	
H2B	0.0196	0.7628	0.3159	0.056*	
N3	0.10939 (15)	0.3376 (2)	0.4093 (2)	0.0615 (6)	
O1	0.08135 (10)	0.53203 (14)	0.77799 (14)	0.0455 (4)	
O2	0.11839 (10)	0.79566 (13)	0.52291 (15)	0.0471 (4)	
O3	0.31267 (10)	0.62292 (17)	0.91261 (15)	0.0547 (4)	
O4	0.35592 (12)	0.82091 (18)	0.85572 (19)	0.0712 (6)	
C1	0.15256 (14)	0.4927 (2)	0.7562 (2)	0.0384 (5)	
C2	0.20453 (13)	0.56267 (19)	0.66513 (19)	0.0349 (5)	
C3	0.28596 (13)	0.4696 (2)	0.6779 (2)	0.0407 (5)	
C4	0.27590 (15)	0.3614 (2)	0.7541 (2)	0.0464 (6)	
C5	0.33919 (17)	0.2580 (2)	0.7802 (3)	0.0633 (7)	
H5	0.3323	0.1857	0.8319	0.076*	
C6	0.41318 (19)	0.2672 (3)	0.7260 (3)	0.0727 (9)	
H6	0.4567	0.1991	0.7413	0.087*	
C7	0.42407 (17)	0.3739 (3)	0.6501 (3)	0.0697 (8)	
H7	0.4749	0.3774	0.6157	0.084*	
C8	0.35978 (15)	0.4768 (3)	0.6242 (2)	0.0528 (6)	
H8	0.3665	0.5487	0.5720	0.063*	
C9	0.30534 (14)	0.7262 (2)	0.8305 (2)	0.0436 (5)	
C10	0.23047 (13)	0.7054 (2)	0.7079 (2)	0.0369 (5)	
C11	0.18724 (14)	0.8094 (2)	0.6392 (2)	0.0405 (5)	
C12	0.19903 (19)	0.9537 (2)	0.6704 (3)	0.0595 (7)	
H12A	0.2337	0.9650	0.7614	0.089*	
H12B	0.1393	0.9945	0.6562	0.089*	
H12C	0.2316	0.9947	0.6139	0.089*	
C13	0.39267 (17)	0.6183 (3)	1.0257 (3)	0.0714 (8)	
H13A	0.4466	0.6437	0.9993	0.107*	
H13B	0.4006	0.5296	1.0608	0.107*	
H13C	0.3843	0.6784	1.0925	0.107*	
C14	0.14114 (13)	0.56297 (19)	0.52501 (19)	0.0344 (5)	
C15	0.10030 (13)	0.67441 (19)	0.4638 (2)	0.0354 (5)	
C16	0.12260 (14)	0.4396 (2)	0.4595 (2)	0.0386 (5)	

### Atomic displacement parameters (Å^2^)

**Table:**

	*U*^11^	*U*^22^	*U*^33^	*U*^12^	*U*^13^	*U*^23^
N1	0.0516 (11)	0.0375 (11)	0.0572 (13)	−0.0057 (9)	0.0114 (9)	0.0099 (9)
N2	0.0498 (11)	0.0354 (10)	0.0468 (12)	−0.0004 (8)	0.0007 (9)	0.0029 (8)
N3	0.0726 (14)	0.0394 (12)	0.0652 (14)	0.0061 (10)	0.0069 (11)	−0.0167 (10)
O1	0.0468 (9)	0.0417 (9)	0.0516 (10)	−0.0092 (7)	0.0195 (7)	−0.0068 (7)
O2	0.0572 (9)	0.0292 (8)	0.0498 (10)	−0.0015 (7)	0.0064 (8)	−0.0020 (7)
O3	0.0523 (9)	0.0615 (11)	0.0430 (10)	−0.0108 (8)	0.0008 (8)	−0.0016 (8)
O4	0.0635 (11)	0.0758 (13)	0.0673 (12)	−0.0367 (10)	0.0062 (9)	−0.0110 (10)
C1	0.0398 (11)	0.0331 (12)	0.0390 (12)	−0.0088 (9)	0.0053 (9)	−0.0050 (9)
C2	0.0345 (10)	0.0324 (11)	0.0363 (11)	−0.0034 (8)	0.0071 (9)	−0.0007 (9)
C3	0.0358 (11)	0.0414 (13)	0.0401 (12)	−0.0010 (9)	0.0022 (9)	−0.0051 (10)
C4	0.0409 (12)	0.0388 (12)	0.0513 (14)	−0.0006 (10)	−0.0014 (10)	−0.0034 (10)
C5	0.0586 (15)	0.0441 (15)	0.0705 (18)	0.0072 (12)	−0.0105 (14)	0.0011 (12)
C6	0.0519 (15)	0.0648 (19)	0.086 (2)	0.0207 (14)	−0.0061 (15)	−0.0161 (16)
C7	0.0434 (14)	0.085 (2)	0.076 (2)	0.0112 (14)	0.0082 (13)	−0.0167 (17)
C8	0.0405 (12)	0.0635 (16)	0.0523 (15)	0.0028 (11)	0.0091 (11)	−0.0049 (12)
C9	0.0409 (11)	0.0503 (14)	0.0415 (13)	−0.0119 (11)	0.0148 (10)	−0.0103 (11)
C10	0.0387 (11)	0.0376 (12)	0.0365 (12)	−0.0106 (9)	0.0140 (9)	−0.0054 (9)
C11	0.0456 (12)	0.0337 (12)	0.0444 (13)	−0.0115 (9)	0.0162 (10)	−0.0070 (10)
C12	0.0803 (17)	0.0336 (13)	0.0654 (17)	−0.0135 (12)	0.0217 (14)	−0.0105 (11)
C13	0.0563 (16)	0.098 (2)	0.0504 (16)	0.0052 (15)	−0.0013 (13)	−0.0040 (15)
C14	0.0353 (10)	0.0304 (11)	0.0372 (12)	−0.0012 (8)	0.0094 (9)	−0.0029 (9)
C15	0.0363 (11)	0.0303 (11)	0.0400 (13)	−0.0037 (8)	0.0110 (10)	−0.0030 (9)
C16	0.0405 (11)	0.0354 (13)	0.0378 (12)	0.0051 (9)	0.0070 (9)	−0.0005 (10)

### Geometric parameters (Å, °)

**Table:**

N1—C1	1.347 (3)	C4—C5	1.385 (3)
N1—C4	1.403 (3)	C5—C6	1.384 (4)
N1—H1	0.8600	C5—H5	0.9300
N2—C15	1.325 (3)	C6—C7	1.372 (4)
N2—H2A	0.8600	C6—H6	0.9300
N2—H2B	0.8600	C7—C8	1.391 (3)
N3—C16	1.146 (3)	C7—H7	0.9300
O1—C1	1.220 (2)	C8—H8	0.9300
O2—C15	1.361 (2)	C9—C10	1.472 (3)
O2—C11	1.372 (3)	C10—C11	1.333 (3)
O3—C9	1.334 (3)	C11—C12	1.489 (3)
O3—C13	1.439 (3)	C12—H12A	0.9600
O4—C9	1.202 (2)	C12—H12B	0.9600
C1—C2	1.558 (3)	C12—H12C	0.9600
C2—C14	1.515 (3)	C13—H13A	0.9600
C2—C3	1.517 (3)	C13—H13B	0.9600
C2—C10	1.523 (3)	C13—H13C	0.9600
C3—C8	1.377 (3)	C14—C15	1.352 (3)
C3—C4	1.382 (3)	C14—C16	1.409 (3)
			
C1—N1—C4	112.07 (18)	C3—C8—C7	118.4 (2)
C1—N1—H1	124.0	C3—C8—H8	120.8
C4—N1—H1	124.0	C7—C8—H8	120.8
C15—N2—H2A	120.0	O4—C9—O3	122.7 (2)
C15—N2—H2B	120.0	O4—C9—C10	126.1 (2)
H2A—N2—H2B	120.0	O3—C9—C10	111.16 (18)
C15—O2—C11	120.13 (15)	C11—C10—C9	120.16 (19)
C9—O3—C13	117.34 (19)	C11—C10—C2	122.26 (18)
O1—C1—N1	126.4 (2)	C9—C10—C2	117.58 (18)
O1—C1—C2	125.59 (19)	C10—C11—O2	122.51 (17)
N1—C1—C2	107.80 (18)	C10—C11—C12	129.5 (2)
C14—C2—C3	111.31 (16)	O2—C11—C12	107.98 (18)
C14—C2—C10	108.97 (16)	C11—C12—H12A	109.5
C3—C2—C10	114.92 (16)	C11—C12—H12B	109.5
C14—C2—C1	107.93 (15)	H12A—C12—H12B	109.5
C3—C2—C1	101.24 (16)	C11—C12—H12C	109.5
C10—C2—C1	112.12 (16)	H12A—C12—H12C	109.5
C8—C3—C4	120.4 (2)	H12B—C12—H12C	109.5
C8—C3—C2	130.7 (2)	O3—C13—H13A	109.5
C4—C3—C2	108.86 (18)	O3—C13—H13B	109.5
C3—C4—C5	121.8 (2)	H13A—C13—H13B	109.5
C3—C4—N1	109.75 (18)	O3—C13—H13C	109.5
C5—C4—N1	128.4 (2)	H13A—C13—H13C	109.5
C6—C5—C4	117.0 (3)	H13B—C13—H13C	109.5
C6—C5—H5	121.5	C15—C14—C16	119.74 (18)
C4—C5—H5	121.5	C15—C14—C2	122.94 (17)
C7—C6—C5	121.8 (3)	C16—C14—C2	117.29 (17)
C7—C6—H6	119.1	N2—C15—C14	128.29 (19)
C5—C6—H6	119.1	N2—C15—O2	110.40 (17)
C6—C7—C8	120.6 (3)	C14—C15—O2	121.30 (18)
C6—C7—H7	119.7	N3—C16—C14	178.0 (2)
C8—C7—H7	119.7		
			
C4—N1—C1—O1	179.4 (2)	O3—C9—C10—C11	153.2 (2)
C4—N1—C1—C2	4.4 (2)	O4—C9—C10—C2	152.4 (2)
O1—C1—C2—C14	−63.3 (2)	O3—C9—C10—C2	−26.1 (3)
N1—C1—C2—C14	111.82 (18)	C14—C2—C10—C11	12.2 (3)
O1—C1—C2—C3	179.74 (19)	C3—C2—C10—C11	137.8 (2)
N1—C1—C2—C3	−5.2 (2)	C1—C2—C10—C11	−107.3 (2)
O1—C1—C2—C10	56.7 (3)	C14—C2—C10—C9	−168.64 (16)
N1—C1—C2—C10	−128.16 (18)	C3—C2—C10—C9	−42.9 (2)
C14—C2—C3—C8	67.4 (3)	C1—C2—C10—C9	71.9 (2)
C10—C2—C3—C8	−57.0 (3)	C9—C10—C11—O2	178.62 (18)
C1—C2—C3—C8	−178.1 (2)	C2—C10—C11—O2	−2.2 (3)
C14—C2—C3—C4	−110.22 (19)	C9—C10—C11—C12	−3.6 (3)
C10—C2—C3—C4	125.30 (19)	C2—C10—C11—C12	175.6 (2)
C1—C2—C3—C4	4.3 (2)	C15—O2—C11—C10	−9.0 (3)
C8—C3—C4—C5	0.6 (3)	C15—O2—C11—C12	172.84 (18)
C2—C3—C4—C5	178.6 (2)	C3—C2—C14—C15	−140.8 (2)
C8—C3—C4—N1	−179.97 (19)	C10—C2—C14—C15	−13.0 (3)
C2—C3—C4—N1	−2.0 (2)	C1—C2—C14—C15	108.9 (2)
C1—N1—C4—C3	−1.6 (2)	C3—C2—C14—C16	41.4 (2)
C1—N1—C4—C5	177.7 (2)	C10—C2—C14—C16	169.18 (17)
C3—C4—C5—C6	−0.4 (3)	C1—C2—C14—C16	−68.8 (2)
N1—C4—C5—C6	−179.6 (2)	C16—C14—C15—N2	0.6 (3)
C4—C5—C6—C7	0.3 (4)	C2—C14—C15—N2	−177.10 (18)
C5—C6—C7—C8	−0.6 (4)	C16—C14—C15—O2	−178.37 (18)
C4—C3—C8—C7	−0.8 (3)	C2—C14—C15—O2	3.9 (3)
C2—C3—C8—C7	−178.3 (2)	C11—O2—C15—N2	−171.11 (16)
C6—C7—C8—C3	0.8 (4)	C11—O2—C15—C14	8.0 (3)
C13—O3—C9—O4	−9.1 (3)	C15—C14—C16—N3	176 (100)
C13—O3—C9—C10	169.40 (19)	C2—C14—C16—N3	−6(7)
O4—C9—C10—C11	−28.4 (3)		

### Hydrogen-bond geometry (Å, °)

**Table:**

*D*—H···*A*	*D*—H	H···*A*	*D*···*A*	*D*—H···*A*
N1—H1···N3^i^	0.86	2.09	2.928 (3)	165
N2—H2A···O1^ii^	0.86	2.17	2.925 (2)	147
N2—H2B···O1^iii^	0.86	2.34	3.022 (2)	136

Symmetry codes: (i) *x*, −*y*+1/2, *z*+1/2; (ii) −*x*, −*y*+1, −*z*+1; (iii) *x*, −*y*+3/2, *z*−1/2.
